# Epidemiology of injuries due to ankle sprain diagnosed in an orthopedic emergency room

**DOI:** 10.31744/einstein_journal/2020AO4739

**Published:** 2019-09-16

**Authors:** Pedro Debieux, Andre Wajnsztejn, Nacime Salomão Barbachan Mansur

**Affiliations:** 1 Hospital Israelita Albert Einstein São PauloSP Brazil Hospital Israelita Albert Einstein, São Paulo, SP, Brazil.; 2 Escola Paulista de Medicina Universidade Federal de São Paulo São PauloSP Brazil Escola Paulista de Medicina, Universidade Federal de São Paulo, São Paulo, SP, Brazil.

**Keywords:** Ankle injuries/etiology, Tarsal bones/injuries, Collateral ligaments, Sprains and strains/epidemiology, Fractures, avulsion, Magnetic resonance imaging, Ankle injuries/epidemiology

## Abstract

**Objective:**

To use magnetic resonance imaging to assess the prevalence of foot and ankle ligament injuries and fractures associated with ankle sprain and not diagnosed by x-ray.

**Methods:**

We included 180 consecutive patients with a history of ankle sprain, assessed at a primary care service in a 12-month period. Magnetic resonance imaging findings were recorded and described.

**Results:**

Approximately 92% of patients had some type of injury shown on the magnetic resonance imaging. We found 379 ligament injuries, 9 osteochondral injuries, 19 tendinous injuries and 51 fractures. Only 14 magnetic resonance imaging tests (7.8%) did not show any sort of injury. We observed a positive relation between injuries of the lateral complex, syndesmosis and medial ligaments. However, there was a negative correlation between ankle ligament injuries and midfoot injuries.

**Conclusion:**

There was a high rate of injuries secondary to ankle sprains. We found correlation between lateral ligament injuries and syndesmosis and deltoid injuries. We did not observe a relation between deltoid and syndesmosis injuries or between lateral ligamentous and subtalar injuries. Similarly, no relation was found between ankle and midfoot injuries.

## INTRODUCTION

Ankle ligament injuries are among the most prevalent traumatic conditions in the emergency room.^( 1 - 3 )^ Albeit widely studied, injuries are generally described jointly as lateral or medial ligament complexes and are rarely individualized. In addition, a patient complaining of ankle sprain may eventually present injuries that affect the midfoot rather than the hindfoot, or indeed the tibiotarsal joint. Historically, articles and publications dealing with ankle sprains overlook the conditions of these regions. Not surprisingly emergency room professionals may, similarly, disregard the diagnosis of these anatomical areas.^( 4 , 5 )^

Depending on the level of energy, ankle and foot position, sprain direction, bone and soft tissue envelope quality, trauma may have different outcomes and diagnoses.^( 6 )^ When appropriately managed, most ankle sprains progress to lateral ligament injuries^( 7 )^ that have good prognosis, and generally do not require many subsidiary tests, which increase costs and delay treatment of patients.^( 5 )^ Such injuries can be diagnosed through a meticulous physical examination, together with good quality radiographs of the segment studied.

Initial clinical assessment takes into account palpation of bone, tendinous, ligamentous and articular structures, as well as performing diagnostic maneuvers, in order to assess the competence of these structures.^( 5 )^ Despite its extreme applicability, this clinical assessment has variable scores of sensitivity and specificity. Van Dijk et al., showed a strong relation between surface anatomy palpation, the presence of hematoma and the positivity of ligament maneuvers on lesions of these structures.^( 8 )^ When performed within the first 48 hours, the physical examination had a sensitivity of 71% and a specificity of 33%. Clinical assessment on the fifth day after trauma showed a sensitivity of 96% and a specificity of 84%, and a positive predictive value of 95% for ligament injury in the association between pain upon palpation of the anterior talofibular ligament (ATFL), presence of hematoma and positive anterior drawer test of ankle. These authors used ankle arthrography as a gold standard to compare findings.^( 8 )^

Magnetic resonance imaging (MRI) in the identification and prognosis of possible injuries associated with ankle sprains has been described since the 1990´s.^( 9 , 10 )^ Some authors have attempted to associate the clinical diagnosis with MRI findings,^( 11 )^ showing a good correlation between the existence of trauma, the clinical classification of sprain severity, and the display of ligament injuries on the MRI.

However, little has been reported on subtalar, midtarsal and tarsometatarsal joint injuries associated with foot and ankle sprain.^( 12 , 13 )^ Thus, injuries of these structures are commonly neglected or underdiagnosed and are usually managed with an empiric, non-specific and ineffective approach.

## OBJECTIVE

To use magnetic resonance imaging to assess the prevalence of different ankle and foot ligament injuries, as well as radiographically non-diagnosable fractures, in patients seeking treatment at the emergency room after ankle sprain.

## METHODS

All patients presenting ankle sprain injury admitted to the Emergency Room of *Hospital Israelita Albert Einstein* (Ibirapuera Unit), from January to December 2015, were assessed and included in the study.

Inclusion criteria were patients of both sexes, aged 8 to 70 years, with an episode of ankle sprain, in any direction (eversion, inversion and combination); and whose radiographs with load had not shown clear signs of fracture, or osteochondral or distal tibiofibular syndesmosis injury. We excluded patients with a previous history of surgery on an injured foot or ankle; history or registered evidence of type 1 or type 2 *diabetes mellitus* (possibility of diabetic peripheral neuropathy); history or registered evidence of systemic inflammatory disease (rheumatoid arthritis, ankylosing spondylitis, Reiter’s syndrome, etc.); pregnancy; impossibility or inability to sign the Informed Consent Form; MRI contraindication; physical or social restriction that would render following the protocol impossible.

After the assessment by history and physical examination performed by the on-call physician, patients with malleolar pain, in the tibiotarsal, midfoot or 5^th^ metatarsal regions, or unable to bear weight on the injured limb were submitted to foot and ankle radiographs in the anteroposterior and lateral views of the foot, and anteroposterior, true anteroposterior and lateral views of the ankle. Patients were submitted to MRI if, on physical examination, they presented pain on ligament insertions, syndesmosis (after provocative maneuvers), midfoot and Lisfranc, and there were no radiographic changes to justify the symptoms. This routine complies with the standards of the hospital unit protocol.

Five to seven days after trauma, an MRI scan was performed at the unit from the lower mid third of the leg to the tarsometatarsal transition of the injured limb, on 1.5 T devices (GE or Siemens), with an ankle dedicated coil (16 channels). Protocol sequences were: sagittal slice in T1, sagittal in T2 with fat saturation, coronal T2 with fat saturation, axial slice in T1, axial T2 with fat saturation, coronal in proton density (PD) oblique (for fibular tendon) and coronal T1, when there was bone edema. As for the thickness of slices, they were 3mm in the sagittal and 3.5mm in the axial and coronal sections - all with 10% gaps.

The same physician performed the patient physical examination and analyzed ankle radiographs. The MRI was assessed by a second physician, a radiologist, specialized in musculoskeletal system. The occurrence of ligament, tendinous, cartilaginous and bony injuries was documented, as well as the relation among them.

The study was approved by the Research Ethics Committee of the *Hospital Israelita Albert Einstein* , opinion 2.664.931, CAAE: 83236118.8.0000.0071.

## RESULTS

During the study period, 244 ankle sprains were assessed. For 180 patients, MRI was indicated complying with the study inclusion and exclusion criteria, and 64 were excluded for not presenting MRI indication or for missing follow up. According to the MRI examination, 92.2% had some type of injury ( Table 1 ). Among the injuries observed, there were 379 ligament injuries (including a Lisfranc injury), 51 foot and ankle fractures (including avulsion fractures), 19 tendon injuries (including a calcaneal tendon injury) and 9 osteochondral injuries (7 at the medial portion of the talus, including one injury due to osteochondritis dissecans). We found 21.7% of patients with some type of fracture. Only 14 MRI exams (7.8%) did not show any type of injury.

**Table 1:** Ligament injuries

Structure	n
Anterior talofibular ligament	135
Calcaneofibular ligament	108
Posterior talofibular ligament	1
Anterior tibiofibular ligament	15
Posterior tibiofibular ligament	1
Superficial deltoid	15
Deep deltoid	63
Dorsal talonavicular	11
Bifurcate ligament	8
Dorsolateral calcaneocuboid	10
Extensor retinaculum	11
Lisfranc	1
Osteochondral injuries	
Medial talus	7
Posterior @tibia	1
Lateral talus	1
Tendinous injuries	
Fibularis brevis	11
Fibularis longus	5
Posterior tibial	1
Osteochondral	9
Extensor digitorum brevis	1
Calcaneal tendon	1

When analyzed by segment, we observed 347 ankle injuries (338 ligamentous and 9 osteochondral), 40 hindfoot injuries and one midfoot injury. Among ligamentous injuries, the most prevalent were ATFL injury with 35.6%, followed by the calcaneofibular ligament (CFL) with 28.5%, and the deep deltoid with 16.9%. The most prevalent osteochondral injury ( Table 1 ) was the medial talus in 77.8%. Among fractures ( Table 2 ), the ATFL avulsion fracture (23.5% of fractures and 6.7% of patients) was the most common, followed by the anterior calcaneus process (17.6% of fractures and 5% of patients) and the cuboid (13.7% of fractures and 3.9% of patients).

**Table 2:** Fracture distribution

Fractures	n
Anterior calcaneal process	9
Avulsion LTFA	12
Lateral malleolus	3
Posterior malleolus	1
CFL avulsion	2
Cuboid	7
Talus	4
Deltoid avulsion	2
Navicular	3
Physeal	1
1^st^ metatarsal bone	2
2^nd^ metatarsal bone	1
3^rd^ metatarsal bone	3
4^th^ metatarsal bone	2

ATFL: anterior talofibular ligament; CFL: calcaneofibular ligament.

**Figure 1:**
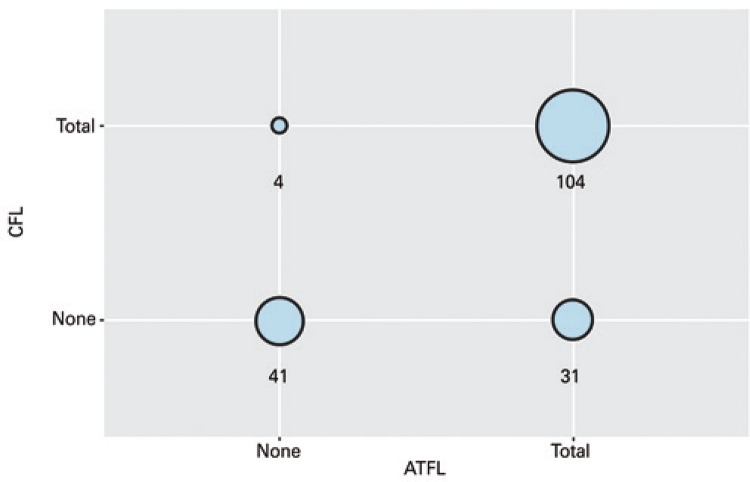
Correlation between categorical variables anterior talofibular ligament (ATFL) and fibulocalcaneal ligament (CFL)

As to associated injuries, the most prevalent association was the ATFL and CFL injury. In 78.5% of cases of ATFL injury, there was an associated CFL injury; in contrast, in 96.3% of cases of CFL injury, there was ATFL injury ( Figure 1 ). Thus, the χ^2^ test checked the association between ATFL and CFL injuries. The p-value of this test was <0.0001, with no evidence that the occurrence of the injury of the two ligaments was independent.

All injuries of the anterior tibiofibular ligament (syndesmosis) were associated with anterior talofibular injuries, with a 73.3% association with CFL injury (p<0.01). Regarding fractures, no correlation was found between any specific ligament injury and an anterior calcaneus process or cuboid fracture (the most prevalent). Of the medial osteochondral injuries, 83.3% were associated with lateral ligament complex injury (excluding an osteochondritis dissecans), and 66.7% were associated with both ATFL and CFL injuries.

When the impact on the treatment of listed injuries was considered, in only one (0.56%) case (talus fracture), conservative treatment was converted to surgery by means of astragalus percutaneous osteosynthesis. All other cases were non-surgical. Regarding the type and time of immobilization, in 15% of cases (27 injuries: 9 osteochondral, 4 ligaments, 8 metatarsal fractures, 3 ankle fractures and one Achilles tendon injury), MRI modified the type of immobilization or the load regimen and early physiotherapy. The χ^2^ test enabled us to measure the correlation among these injuries ( Tables 3 to 5 [Table t4] [Table t5] ).

**Table 3:** Ligament complex and co-variables correlation

	Lateral ligament complex			p value

	No	Yes	Total	
	n (%)	n (%)	n (%)	
Deltoid				<0.001*
No	36 (95)	78 (55)	114 (64)	
Yes	2 (5)	63 (45)	65 (36)	
Syndesmosis				0.063*
No	38 (100)	125 (89)	163 (91)	
Yes	0 (0)	16 (11)	16 (9)	
Subtalar				0.243*
No	29 (76)	119 (84)	148 (83)	
Yes	9 (24)	22 (16)	31 (17)	

* Yates correlation.

**Table 4 t4:** Syndesmosis and co-variables correlation

	Syndesmosis			p value

	No	Yes	Total	
	n (%)	n (%)	n (%)	
Deltoid				0.324*
No	102 (63)	12 (75)	114 (64)	
Yes	61 (37)	4 (25)	65 (36)	
Subtalar				0.220*
No	133 (82)	15 (94)	148 (83)	
Yes	30 (18)	1 (6)	31 (17)	

* Yates correlation.

**Table 5 t5:** Deltoid and subtalar correlation

	Deltoide			p value
	
	No	Yes	Total	
	n (%)	n (%)	n (%)	
Deltoid				0.916*
No	94 (82)	54 (83)	148 (83)	
Yes	20 (18)	11 (17)	31 (17)	
Total	114 (64)	65 (36)	179 (100)	

* Yates correlation.

There was a relation between lateral ligament complex injury and medial deltoid injury, as well as syndesmosis. In both scenarios, we had combinations in less than five cases, and it was necessary to perform correction using Yates. Even after correction, relations remained significant. Thus, patients with lateral ligament injuries tended to also have deltoid and syndesmosis injuries. Conversely, patients who did not have a lateral ligament injury were also not likely to present deltoid and syndesmosis injuries.

No correlation was observed between ligament injuries and any of the fractures seen in this series either. On the other hand, there was a negative association between lateral ligament complex injury and midfoot ligament injuries: when one injury was present, no injury was observed on the other.

## DISCUSSION

The study aimed to describe the injuries related to ankle sprains treated at a general orthopedic emergency room, report underdiagnosed injuries, establish the diagnostic investigation relevance of complementary imaging tests when the criteria for such are met and, finally to elucidate relations between injuries, in order to create a ligament and bone injury ranking, using only prevalence criteria. Such ranking could minimize diagnosis omission and reduce the risk of inappropriate treatment.

The core of ankle sprain injury diagnosis orbits around the lateral ligament complex. Not only ATFL injury is the most prevalent, but it also has high correlation with several ankle lateral ligament injuries, in addition to lateral complex injuries related to medial structures and syndesmosis. On the other hand, lateral injuries present negative correlation with midfoot injuries. This is corroborated by the biomechanical and kinematic analysis of the ankle sprain, since trauma energy will dissipate at another point.^( 14 - 17 )^

The second relevant finding is the prevalence of fractures in ankle sprains. We reported 52 fractures in 180 patients. Some patients had more than one fracture, and 21.7% of patients with ankle sprains presented bone injury. Two reflections essentially emerge from the data. Knowing all injuries were diagnosed by MRI and none by radiographs, one can initially raise the question on ankle plain film efficiency. In this context, it was clear that, with the exception of one patient, no fracture had a significant deviation or fracture line that would justify management change. Thus, instead of being attributed to the lack of radiographic sensitivity, one can question the need for MRI testing and its higher sensitivity in this type of diagnosis, since there was no interference of the test in the management chosen. It is worth mentioning that there was conversion to surgical treatment for one patient (talus fracture).^( 18 , 19 )^

In contrast, we can conclude that when there are midfoot injury signs, either by trauma mechanism or clinical presentation, further investigation with imaging tests may be recommended, especially when there is no suspected lateral ligament complex injury. This is not only due to the association of midfoot injuries with absence of lateral complex injuries, but also to the low sensitivity of the radiographs for foot and ankle fractures in general. It is evident, however, that additional investigation is more linked to the anxiety for a precise diagnosis than to any effect on outcome.

Unlike MRI, there is consensus about radiograph request. Due to the significant number of visits, clinical criteria were defined for performing radiographs on patients suffering from ankle sprain. Indiscriminate use of radiographs contributes to an increase in cost and time of treatment, as well as exposing patients to ionizing radiation. The most commonly used criteria are the Ottawa rules (pain in the malleolus or midfoot, pain in the base of the fifth metatarsal or navicular, edema in these regions, impossibility to take four steps after the injury, or at the moment of visit).^( 20 , 21 )^

Beckenkamp et al., performed a systematic review on the accuracy of the Ottawa rules and concluded that these criteria are highly sensitive (99.4%) to rule out visible fractures on plain films, despite low specificity (35.3%) and exposure of several patients to radiation with normal radiographs.^( 22 )^

When focusing on treatment, there is no consensus on management of acute ankle sprain. Doherty et al., reviewed and concluded that there is strong evidence indicating use of non-steroidals anti-inflammatory drugs and early mobilization. Exercises and manual physical therapy are recommended to improve pain, edema and function. Exercise and immobilization are recommended to avoid recurrence of symptoms and to decrease risk of chronic ankle instability.^( 23 )^

In a background of disagreement regarding the need for high cost tests to investigate a highly prevalent injury in an emergency care environment, understanding the behavior of foot and ankle stabilizers and establishing their epidemiological characteristics are mandatory. This understanding would reduce divergences regarding the best treatment progress from immobilization to functional treatment, including even more aggressive management, such as ligament repair and reconstruction, to prevent chronic instability and its consequences,

The study presents limitations, mostly related to its design. The most important limitation was the absence of correlation between clinical presentation and imaging, without follow-up or clinical outcomes. Although the number of participants was insufficient for certain statistical tests, the Yates correlation could be used. Magnetic resonance imaging was performed only on patients with indication, according to the institutional protocol, following the guidelines established by the Ethics Committee. This explains the high rate of ligament injury found.

## CONCLUSION

The most prevalent injuries in ankle sprains were the lateral ligament complex and deltoid ligament complex. The most prevalent fractures were avulsions of the calcaneus anterior process, fibula and cuboid.

The prevalence of underdiagnosed injuries associated with ankle sprain, such as tendinous (fibular) and osteochondral injuries and midfoot fractures, were high in the orthopedic emergency room.

Patients with persistent complaints and positive physical examination on unusual sites may benefit from magnetic resonance imaging to establish better diagnoses and appropriate management of injuries.
