# Supplementary material for: Epidemiology of injuries due to ankle sprain diagnosed in an orthopedic emergency room
Source: Einstein (Sao Paulo). 2019 Sep 16;18:eAO4739. doi: 10.31744/einstein_journal/2020AO4739 (PMC6905160; doi:10.31744/einstein_journal/2020AO4739)
Supplement: Translation

# Translation: Epidemiologia das lesões por entorse do tornozelo diagnosticadas em pronto atendimento de ortopedia

**Authors:** Pedro Debieux, Andre Wajnsztejn, Nacime Salomão Barbachan Mansur

**Affiliations:** 1 Brasil Hospital Israelita Albert Einstein, São Paulo, SP, Brasil.; 2 Brasil Escola Paulista de Medicina, Universidade Federal de São Paulo, São Paulo, SP, Brasil.

**Keywords:** Traumatismos do tornozelo/etiologia, Ossos do tarso/lesões, Ligamentos colaterais, Entorses e distensões/epidemiologia, Fratura avulsão, Imagem por ressonância magnética, Traumatismos do tornozelo/epidemiologia

## Abstract

**Objetivo:**

Avaliar na ressonância magnética a prevalência das diferentes lesões ligamentares do tornozelo e do pé, bem como de fraturas não diagnosticáveis radiograficamente, em pacientes com queixa de entorse do tornozelo.

**Métodos:**

Foram incluídos no estudo 180 pacientes consecutivos, com história de entorse do tornozelo, atendidos em um serviço de Atenção Primária no período de 12 meses. Os achados dos exames de ressonância magnética foram catalogados e descritos.

**Resultados:**

Aproximadamente 92% dos pacientes apresentaram algum tipo de lesão na ressonância. Dentre as injúrias observadas, estavam 379 lesões ligamentares, 9 lesões osteocondrais, 19 lesões tendíneas e 51 fraturas. Apenas 14 ressonâncias magnéticas (7,8%) não mostraram qualquer tipo de lesão. Observamos relação positiva entre lesões do complexo lateral, sindesmose e medial. No entanto, houve correlação negativa entre lesões ligamentares do tornozelo e aquelas do mediopé.

**Conclusão:**

Foi alta a ocorrência de lesões secundárias à entorse. Apontamos correlação entre as lesões ligamentares laterais com as sindesmodais e do deltoide. Não notamos relação entre as lesões do deltoide e da sindesmose, e nem entre as ligamentares laterais e a subtlalar. Também não foram observadas relações entre as lesões do tornozelo e as do mediopé.

## INTRODUÇÃO

As lesões ligamentares do tornozelo estão entre as afecções traumáticas mais prevalentes em ambiente de pronto atendimento.^( 1 - 3 )^Apesar de amplamente estudadas, as lesões são, em geral, descritas conjuntamente como complexos ligamentares lateral ou medial, raramente sendo individualizadas. Adicionalmente, um paciente que se queixa de entorse no tornozelo pode, eventualmente, apresentar lesões que acometam mais o mediopé do que o retropé, ou a articulação tibiotársica de fato. Historicamente, artigos e publicações que tratam de entorses negligenciam as afecções destas regiões. Neste contexto, o profissional em pronto atendimento pode, semelhantemente, ignorar diagnósticos destas áreas anatômicas.^( 4 , 5 )^

Dependendo do grau de energia, da posição do tornozelo e do pé, da direção da entorse, da qualidade óssea e do envelope de tecidos moles, esse trauma pode ter diferentes desfechos e diagnósticos.^( 6 )^Em sua maioria, essas entorses evoluem para lesões ligamentares laterais do tornozelo,^( 7 )^que tem bom prognóstico, quando bem conduzidas, e, geralmente, não requerem muitos exames subsidiários, que encarecem e retardam o tratamento dos pacientes.^( 5 )^Tais injúrias podem ser diagnosticadas por meio de um minucioso exame físico, aliado à realização de boas radiografias do segmento estudado.

A avaliação clínica inicial leva em consideração a palpação de estruturas ósseas, tendíneas, ligamentares e articulares, além da realização de manobras semióticas, para aferição da competência dessas estruturas.^( 5 )^Apesar de sua extrema aplicabilidade, essa análise tem índices de sensibilidade e especificidade variados. Van Dijk et al., mostraram alta relação entre a palpação da anatomia de superfície, a presença de hematoma e a positividade de testes ligamentares com as lesões dessas estruturas.^( 8 )^Quando realizado nas primeiras 48 horas, o exame físico apresenta sensibilidade de 71% e especificidade de 33%. A avaliação clínica no quinto dia após o trauma demonstrou sensibilidade de 96% e especificidade de 84%, sendo o valor preditivo positivo de lesão ligamentar de 95% na associação entre dor à palpação do ligamento talofibular anterior (LTFA), presença de hematoma e a positividade do teste da gaveta anterior do tornozelo. Esses autores utilizaram a artrografia do tornozelo como padrão-ouro na comparação dos achados.^( 8 )^

A utilização da ressonância magnética (RM) na identificação e no prognóstico das possíveis lesões associadas a uma entorse do tornozelo é descrita desde a década de 1990.^( 9 , 10 )^Alguns autores tentaram associar o diagnóstico clínico aos achados desse exame de imagem,^( 11 )^mostrando boa correlação entre a existência do trauma, a classificação clínica da gravidade da entorse e a ocorrência de lesões ligamentares na RM.

Porém, pouco tem sido relatado em relação às lesões da articulação subtalar, mediotársica e tarsometatársicas na ocorrência de entorses do pé e tornozelo.^( 12 , 13 )^Assim, as lesões destas partes são comumente negligenciadas ou subdiagnosticadas, sendo costumeiramente manejadas de maneira empírica, inespecífica e pouco efetiva.

## OBJETIVO

Avaliar, pela ressonância magnética, a prevalência das diferentes lesões ligamentares do tornozelo e pé, bem como de fraturas não diagnosticáveis radiograficamente, em pacientes que procuram o pronto atendimento com queixa de entorse do tornozelo.

## MÉTODOS

Todos os pacientes vítimas de entorse do tornozelo que deram entrada no Pronto Atendimento do Hospital Israelita Albert Einstein (Unidade Avançada Ibirapuera) durante o período de janeiro a dezembro de 2015 foram avaliados e incluídos no estudo.

Os critérios de inclusão foram pacientes de 8 a 70 anos, de ambos os sexos; com episódio de entorse do tornozelo, em qualquer direção (eversão, inversão e combinação); e cujas radiografias com carga não tinham sinais claros de fraturas, lesões osteocondrais e da articulação da sindesmose tibiofibular distal. Foram excluídos pacientes com história pregressa de cirurgia no pé ou tornozelo acometido; história ou evidência documentada de *diabetes mellitus* tipos 1 ou 2 (possibilidade de neuropatia periférica diabética); história ou evidência documentada de doença inflamatória sistêmica (artrite reumatoide, espondilite anquilosante, síndrome de Reiter etc.); gravidez; impossibilidade ou incapacidade de assinar o Termo de Consentimento Livre e Esclarecido ou qualquer contraindicação à RM; qualquer limitação física ou social que impossibilitasse o seguimento do protocolo.

Após avaliação por história e exame físico do médico plantonista, os pacientes que apresentavam dor maleolar, na tibiotársica, no mediopé ou no quinto metatarso, ou, ainda, incapacidade de descarga de peso no membro acometido, foram submetidos a radiografias do pé e tornozelo nas incidências anteroposterior e perfil do pé, além de anteroposterior, anteroposterior verdadeiro e perfil do tornozelo. Uma vez que o paciente apresentasse, ao exame físico, dor nas inserções ligamentares, sindesmose (ou manobras provocantes), mediopé e Lisfranc, e não possuísse alterações radiográficas que as justificassem, ele era, então, submetido ao exame de RM. Essa rotina obedeceu aos princípios propostos no protocolo institucional da unidade.

Os exames de RM foram realizados do terço médio inferior da perna até a transição tarsometatársica dos membros afetados, na unidade, em 5 a 7 dias após a lesão, em aparelhos de 1,5 T (GE ou Siemens), com bobina específica para tornozelo (16 canais). As sequências preconizadas em protocolo foram: corte sagital em T1, sagital em T2 com saturação de gordura, coronal T2 com saturação de gordura, corte axial em T1, axial T2 com saturação de gordura, coronal em densidade de prótons (DP) oblíquo (para tendão fibular) e coronal T1, quando houvesse edema ósseo. Quanto à espessura dos cortes, foram de 3mm nos sagitais e 3,5mm nos axiais e coronais − todos com *gaps* de 10%.

O mesmo médico executou avaliação do paciente por exame físico e radiografias. A RM foi avaliada por um segundo médico, radiologista, especializado no sistema musculoesquelético. A ocorrência das lesões ligamentares, tendíneas, cartilagíneas e ósseas foi documentada, assim como a relação entre as mesmas.

O estudo foi aprovado pelo Comitê de Ética e Pesquisa do Hospital Israelita Brasileira Albert Einstein, sob o parecer 2.664.931, CAAE: 83236118.8.0000.0071.

## RESULTADOS

Durante o período do estudo, foram avaliadas 244 entorses do tornozelo. Destas, 180 pacientes tiveram indicação de RM e preencheram os critérios de inclusão e exclusão do estudo. Assim, 64 pacientes foram excluídos por não terem indicação do exame ou por perda de seguimento. De acordo com o exame de RM, 92,2% apresentavam algum tipo de lesão ( Tabela 1 ). Dentre as lesões observadas, incluíram-se 379 lesões ligamentares (incluindo uma lesão de Lisfranc), 51 fraturas de pé e tornozelo (incluindo fraturas por avulsão), 19 lesões tendíneas (incluindo uma lesão do tendão calcâneo) e 9 lesões osteocondrais (sendo 7 da região medial do tálus, incluindo uma lesão por osteocondrite dissecante). Encontramos 21,7% dos pacientes com algum tipo de fratura. Apenas 14 RM (7,8%) não mostraram nenhum tipo de lesão.

**Tabela 1:** Lesões ligamentares

Topografia	n
LTFA	135
LFC	108
LTFP	1
TFA	15
TFP	1
Deltóide superficial	15
Deltóide profundo	63
Talo-navicular dorsal	11
Bifurcado	8
Calcâneo-cubóide dorso-lat	10
Retináculo extensores	11
Lisfranc	1
Lesões osteocondrais	
Tálus medial	7
Tíbia posterior	1
Tálus lateral	1
Lesões tendíneas	
Fibular curto	11
Fibular longo	5
Tibial posterior	1
Extensor curto dos dedos	1
Tendão calcâneo	1

Dividindo-se por segmento, foram observadas 347 lesões no tornozelo (338 ligamentares e 9 osteocondrais), 40 lesões no retropé e uma no mediopé. Dentre as lesões ligamentares, a mais prevalente foi a lesão do LTFA com 35,6%, seguida do ligamento fibulocalcâneo (LFC), com 28,5%, e do deltoide profundo, com 16,9%. A lesão osteocondral mais prevalente ( Tabela 1 ) foi a medial do tálus em 77,8%. Já dentre as fraturas ( Tabela 2 ), observou-se que a mais comum foi a fratura avulsão do LTFA (23,5% das fraturas e 6,7% dos pacientes), seguida do processo anterior do calcâneo (17,6% das fraturas e 5% dos pacientes) e do cuboide (13,7% das fraturas e 3,9% dos pacientes).

**Tabela 2:** Ocorrência de fraturas

Fraturas	n
Processo anterior calcâneo	9
Avulsão LTFA	12
Maléolo lateral	3
Maléolo posterior	1
Avulsão LCF	2
Cuboide	7
Tálus	4
Avulsão deltoide	2
Navicular	3
Fisaria	1
Primeiro metatarso	2
Segundo metatarso	1
Terceiro metatarso	3
Quarto metatarso	2

LTFA: ligamento talofibular anterior; LCF: ligamento fibulocalcâneo.

Quanto às lesões associadas, a associação mais prevalente foi a lesão do LTFA e do LFC. Em 78,5% dos casos de lesão do LTFA, houve lesão do LFC associada; em oposição, em 96,3% dos casos de lesão do LFC, houve lesão do LTFA ( Figura 1 ). Assim, aplicou-se o teste χ^2^para verificar a associação entre as lesões do LTFA e LFC. O valor de p desse teste foi <0,0001, sem evidência de que a ocorrência de lesão nos dois ligamentos fosse independente.

**Figura 1:** Correlação entre as variáveis categóricas ligamento talofibular anterior (LTFA) e ligamento fibulocalcâneo (LFC)

Todas as lesões do ligamento tibiofibular anterior (sindesmose) foram associadas à lesões do talofibular anterior, havendo ainda 73,3% de associação com a lesão do LFC (p<0,01). Quanto às fraturas, não se observou correlação entre a fratura do processo anterior do calcâneo ou do cuboide (as mais prevalentes) com qualquer lesão ligamentar específica. Das lesões osteocondrais mediais, 83,3% tiveram associação com lesão do complexo ligamentar lateral (excluindo-se uma osteocondrite dissecante), sendo que 66,7% foram associadas com lesões tanto do LTFA quanto do LFC.

Quando se considerou o impacto no tratamento das lesões listadas, em apenas um (0,56%) caso (fratura do tálus), houve conversão do tratamento conservador para cirúrgico, por meio da osteossíntese percutânea do astrágalo. Todos os demais casos mantiveram-se em tratamento não operatório. Em relação ao tipo e ao tempo de imobilização, em 15% dos casos (27 lesões: 9 osteocondrais, 4 ligamentares, 8 fraturas de metatarsos, 3 fraturas do tornozelo e uma lesão do tendão de Aquiles) a RM modificou o tipo de imobilização ou o regime de carga e fisioterapia precoce. Por meio do teste χ^2^, pudemos aferir a correlação entre essas lesões ( Tabelas 3 a 5 [Table t4002] [Table t5002] ).

**Tabela 3:** Relação de complexo ligamentar lateral com covariáveis

	Ligamentar lateral			Valor de p
	
	Não	Sim	Total	
	n (%)	n (%)	n (%)	
Deltoide				<0,001*
Não	36 (95)	78 (55)	114 (64)	
Sim	2 (5)	63 (45)	65 (36)	
Sindesmose				0,063*
Não	38 (100)	125 (89)	163 (91)	
Sim	0 (0)	16 (11)	16 (9)	
Subtalar				0,243*
Não	29 (76)	119 (84)	148 (83)	
Sim	9 (24)	22 (16)	31 (17)	

* Correlação de Yates.

**Tabela 4 t4002:** Relação de sindesmose com covariáveis

	Sindesmose			Valor de p

	Não	Sim	Total	
	n (%)	n (%)	n (%)	
Deltoide				0,324*
Não	102 (63)	12 (75)	114 (64)	
Sim	61 (37)	4 (25)	65 (36)	
Subtalar				0,220*
Não	133 (82)	15 (94)	148 (83)	
Sim	30 (18)	1 (6)	31 (17)	

* Correlação de Yates.

**Tabela 5 t5002:** Relação de deltoide com subtalar

	Deltoide			Valor de p
	
	Não	Sim	Total	
	n (%)	n (%)	n (%)	
Deltoide				0,916*
Não	94 (82)	54 (83)	148 (83)	
Sim	20 (18)	11 (17)	31 (17)	
Total	114 (64)	65 (36)	179 (100)	

* Correlação de Yates.

Houve relação entre a lesão do complexo ligamentar lateral com a lesão medial do deltoide, bem como da sindesmose. Em ambos os casos, temos combinações com menos de cinco casos, tendo sido necessário realizar a correção de Yates. Mesmo após a correção, as relações continuaram significantes. Assim, pacientes com lesão ligamentar lateral tenderam a ter também as lesões de deltoide e sindesmose. Já as pessoas que não tinham lesão ligamentar lateral tenderam também a não terem estas lesões.

Foi ainda observada ausência de correlação entre as lesões ligamentares e qualquer das fraturas vistas nesta série. Por outro lado, houve associação negativa entre lesão do complexo ligamentar lateral e lesões ligamentares do médio pé: quando houve lesão de um, não se observou lesão do outro.

## DISCUSSÃO

Este estudo buscou estabelecer o *status* das leões por entorse de tornozelo que chegaram a um pronto atendimento de ortopedia geral, relacionar as lesões subdiagnosticadas, estabelecer a importância de exames de imagem adicionais na investigação diagnóstica quando há critérios para tal e, por fim, elucidar relações entre as lesões, de forma a criar uma hierarquia de lesões ligamentares (e ósseas), por critérios unicamente de prevalência, no intuito de minimizar perdas diagnósticas, reduzindo o risco de tratamentos inadequados.

A essência do diagnóstico das lesões por entorse gira ao redor do complexo ligamentar lateral. Não apenas a lesão do LTFA é a mais prevalente, como guarda estreita correlação com outras lesões ligamentares laterais do tornozelo, além das lesões do complexo lateral relacionadas às estruturas mediais e mesmo à sindesmose. Por outro lado, a lesão lateral apresenta correlação negativa com lesões do médio pé. Este dado é corroborado, no raciocínio biomecânico da cinética do entorse, uma vez que a energia do trauma se dissipará em outro ponto.^( 14 - 17 )^

O segundo tópico de menção necessária é a prevalência de fraturas nas entorses de tornozelo. Foram contabilizadas 52 fraturas em 180 pacientes. Alguns pacientes tiveram mais que uma fratura, o que leva ao número de 21,7% dos pacientes com entorse do tornozelo terem algum tipo de envolvimento ósseo. Deste dado desprendem-se essencialmente duas reflexões. Sabendo-se que todas essas lesões foram diagnosticadas por RM e nenhuma delas por radiografias, pode-se, inicialmente, levantar-se a questão a respeito da eficiência da radiografia comum. Neste contexto, foi nítido que, com exceção de um paciente, nenhuma das fraturas teve desvio significativo ou traço que justificasse alteração de conduta. Dessa maneira, ao invés de atribuir à falta de sensibilidade radiográfica, pode-se questionar a necessidade do exame de RM e sua supersensibilidade neste tipo de diagnóstico, uma vez que não houve interferência deste método na conduta adotada. Vale mencionar que, em um caso, houve conversão para o tratamento cirúrgico (fratura do tálus).^( 18 , 19 )^

Em oposição, podemos concluir que, quando há sinais de lesão do médio pé, seja pelo mecanismo de trauma ou pela clínica apresentada, a investigação adicional com exames de imagem pode ser aplicada, especialmente quando não houver suspeita de lesão do complexo ligamentar lateral. Isso porque não apenas constatou-se a associação de lesões do médio pé com a ausência de lesões do complexo lateral, mas também foi observada a baixa sensibilidade do exame radiográfico para fraturas do pé e tornozelo em geral. É nítido, contudo, que esta investigação adicional está mais vinculada à ansiedade do diagnóstico preciso do que a qualquer efeito no desfecho.

Diferentemente da RM, a radiografia possui consenso sobre sua necessidade. Devido ao número expressivo de atendimentos, foram definidos critérios clínicos para a realização de exames radiográficos em pacientes que sofrem entorse do tornozelo. O uso indiscriminado de exames de radiografias leva ao aumento do custo e do tempo de atendimento, além de expor os doentes a um exame com radiação ionizante. O critério mais utilizado são as regras de Ottawa (dor nos maléolos ou no mediopé, dor na base do quinto metatarso ou navicular, edema nestas regiões, impossibilidade de dar quatro passos após a lesão ou no momento do atendimento).^( 20 , 21 )^

Beckenkamp et al., fizeram revisão sistemática sobre a acurácia das regras de Ottawa e concluíram que estes critérios apresentam grande sensibilidade (99,4%) em excluir as fraturas visíveis em radiografias simples, apesar de baixa especificidade (35,3%) e de expor muitas pessoas com radiografias normais a radiação.^( 22 )^

Quando o enfoque é o tratamento, não há consenso sobre a conduta no entorse agudo do tornozelo. Doherty et al., fizeram revisão e concluíram que existem fortes evidências que indicam o uso de anti-inflamatórios não hormonais e mobilização precoce. Para a melhora da dor, edema e função, recomendam-se exercícios e terapias manuais. Exercícios e imobilização são recomendados para evitar a recorrência dos sintomas e para diminuir o risco de uma instabilidade crônica do tornozelo.^( 23 )^

Em um contexto de desacordo em relação à necessidade de exames de alto custo para investigação de uma injúria altamente prevalente em ambiente de pronto atendimento, compreender o comportamento dos estabilizadores do pé e tornozelo é mandatório, assim como estabelecer suas características epidemiológicas. Esse entendimento diminuiria divergências quanto a melhor conduta, que transita da imobilização ao tratamento funcional, incluindo até mesmo tratamentos mais agressivos, como os reparos e as reconstruções ligamentares, a fim de evitar a instabilidade crônica e suas consequências,

Este estudo teve limitações. A maior parte delas foi inerente ao seu desenho. A principal delas foi a ausência de correlação clínica com os exames de imagem, sem seguimento ou desfechos clínicos. Quanto ao número de participantes, embora tenha sido insuficiente para certos testes estatísticos, pôde-se utilizar a correlação de Yates. O exame de RM não foi aplicado em todos os pacientes, somente naqueles com indicação, de acordo com protocolo institucional, seguindo preceitos estabelecidos pelo Comitê de Ética. Isso justifica o alto índice de lesões ligamentares encontradas.

## CONCLUSÃO

As lesões mais prevalentes nos entorses de tornozelo foram ligamentares do complexo lateral e do complexo deltoide. As fraturas mais prevalentes foram avulsões do processo anterior do calcâneo, da fíbula e do cuboide.

Foi alta a prevalência de lesões secundárias à entorse subdiagnosticada no pronto atendimento de ortopedia, como lesões tendíneas (fibulares) e osteocondrais, além de fraturas do mediopé.

Pacientes com persistência de queixa e exame físico positivo em locais não habituais podem se beneficiar da ressonância magnética a fim de que o diagnóstico seja melhor estabelecido e a conduta, direcionada para tal.
